# Die Datenintegrationszentren – Von der Konzeption in der Medizininformatik-Initiative zur lokalen Umsetzung in einem Netzwerk Universitätsmedizin

**DOI:** 10.1007/s00103-024-03879-5

**Published:** 2024-04-25

**Authors:** Fady Albashiti, Reinhard Thasler, Thomas Wendt, Franziska Bathelt, Ines Reinecke, Björn Schreiweis

**Affiliations:** 1 Zentrum für Medizinische Datenintegration und -analyse (MeDICLMU), LMU Klinikum, München, Deutschland; 2 Datenintegrationszentrum, Medizininformatikzentrum, Universitätsklinikum Leipzig AöR, Leipzig, Deutschland; 3 Datenintegrationszentrum, Thiem-Research GmbH, Carl-Thiem-Klinikum Cottbus gGmbH, Cottbus, Deutschland; 4 Datenintegrationszentrum, Zentrum für Medizinische Informatik, Universitätsklinikum Carl Gustav Carus Dresden, Dresden, Deutschland; 5 Institut für Medizinische Informatik und Statistik (IMIS), Christian-Albrechts-Universität zu Kiel & Universitätsklinikum Schleswig-Holstein, Kiel, Deutschland; 6 Zentrum für Medizinische Datenintegration und -analyse, LMU Klinikum, Fraunhoferstr. 20, 82152 Martinsried (Planegg), Deutschland

**Keywords:** Routinedaten, Digitalisierung, Datenintegration, Forschungsdaten-Dienstleistungen, Realworld Data, Digitalization, Data integration, Health research data services

## Abstract

Im Rahmen der Medizininformatik-Initiative (MII) sind seit 2018 an 38 universitären sowie 3 nichtuniversitären Standorten in Deutschland Datenintegrationszentren (DIZ) entstanden. Hier werden Forschungs- und Versorgungsdaten zusammengetragen. Die Datenintegrationszentren (DIZ) stellen mittlerweile eine wichtige Säule in der Forschung und Versorgung dar. Sie schaffen die technischen, organisatorischen und (ethisch-)datenschutzrechtlichen Voraussetzungen, um mit den vorhandenen klinischen Routinedaten auch standortübergreifende Forschung zu ermöglichen.

In diesem Beitrag werden die 3 Hauptsäulen der DIZ vorgestellt: ethisch-rechtlicher Rahmen, Organisation und Technik. Die Organisation von DIZ sowie deren organisatorische Einbettung und Interaktion werden vorgestellt ebenso wie die technische Infrastruktur. Die Services, die ein DIZ für den eigenen Standort und für externe Forschende erbringt, werden erklärt und die Rolle des DIZ als Schnittstelle nach innen und außen zur Stärkung der Kooperation und Kollaboration dargelegt.

Rechtskonformität, Organisation und Technik bilden die Grundlagen für Prozesse und Strukturen eines DIZ und sind einerseits entscheidend dafür, wie es in die Versorgungs- und Forschungslandschaft eines Standortes integriert ist, andererseits aber auch dafür, wie es auf nationale und europäische Anforderungen reagieren und als Schnittstelle nach außen agieren und fungieren kann. In diesem Kontext und im Hinblick auf die nationalen Entwicklungen (z. B. Einführung der elektronischen Patientenakte – ePA), aber auch die internationalen und europäischen Initiativen (z. B. Europäischer Gesundheitsdatenraum – EHDS) werden die DIZ zukünftig eine zentrale Rolle spielen.

## Abstract (en)

As part of the Medical Informatics Initiative (MII), data integration centers (DICs) have been established at 38 university and 3 non-university locations in Germany since 2018. At DICs, research and healthcare data are collected. The DICs represent an important pillar in research and healthcare. They establish the technical, organizational, and (ethical) data protection requirements to enable cross-site research with the available routine clinical data.

This article presents the three main pillars of DICs: ethical–legal framework, organization, and technology. The organization of DICs and their organizational embedding and interaction are presented, as well as the technical infrastructure. The services that a DIC provides for its own location and for external researchers are explained, and the role of the DIC as an internal and external interface for strengthening cooperation and collaboration is outlined.

Legal conformity, organization, and technology form the basis for the processes and structures of a DIC and are decisive for how it is integrated into the healthcare and research landscape of a location, but also for how it can react to national and European requirements and act and function as an interface to the outside world. In this context and with regard to national developments (e.g., introduction of the electronic patient file—ePA), but also international and European initiatives (e.g., European Health Data Space—EHDS), the DIC will play a central role in the future.

## Einleitung

Mit der zunehmenden Digitalisierung des Klinikalltags entstehen große Mengen an Daten. Diese Daten unterscheiden sich in ihrem Umfang (Volume), in der Geschwindigkeit, in der diese erzeugt und übertragen werden (Velocity), und in der Bandbreite der Datenquellen, -typen und Kategorien (Variety; [1]). Um diese Daten aus der Versorgung besser nutzbar zu machen, werden Infrastrukturen gebraucht, die sowohl entsprechende technische als auch organisatorische und (datenschutz-)rechtliche Rahmenbedingungen schaffen [2]. Gleiches gilt für Daten aus der Forschung, insbesondere zu experimentellen Behandlungen, wie etwa neuen Formen der Immuntherapie bei Krebserkrankungen.

Die Aufgabe der Nutzbarmachung dieser Daten haben die im Rahmen der Medizininformatik-Initiative (MII) seit 2018 etablierten Datenintegrationszentren (DIZ) übernommen. Sie werden als Infrastruktur im Netzwerk Universitätsmedizin (NUM) mit dem Ziel der Verstetigung weiter gefördert. Die MII verfolgt das Ziel, die klinischen Routinedaten aus der medizinischen Versorgung für die medizinische Forschung verfügbar und nutzbar zu machen und damit Forschungsprojekte zu ermöglichen, um Therapie‑, Diagnose- und Präventionsmöglichkeiten zu verbessern [3]. Die Aktivitäten des NUM zielen auf die Etablierung eines bundesweiten Studien- und Datenraumes, die Schaffung einer zentralen Anlaufstelle als Ansprechpartner zu klinischer Forschung und auf die Vorbereitung der Versorgungs- und Forschungslandschaft auf zukünftige Pandemien und Krisen [4]. An der MII und dem NUM beteiligen sich die Standorte der Universitätsmedizin in Deutschland sowie weitere. Die DIZ stehen Forschungsprojekten beider Initiativen gleichberechtigt zur Verfügung und sind darüber hinaus in weitere Forschungsprojekte eingebunden. An ihren jeweiligen Standorten treiben die DIZ die datengetriebene Forschung und Versorgung voran und beschleunigen die Translation von Forschungsergebnissen in die Versorgung [5]. Sie erschließen dazu lokal fortlaufend neue Datenquellen und integrieren klinische Routinedaten bspw. mit Bioprobendaten aus Forschungsbiobanken. Dabei setzten sie die in der MII angeforderten Standards und definierten Spezifikationen um. Sie fungieren darüber hinaus als Ideen- und Impulsgeber für Digitalisierungsmöglichkeiten von Arbeits- und Dokumentationsprozessen und tragen so zu einer optimierten Versorgung und verbesserten Datenqualität bei.

Dabei sind die Herausforderungen einer umfassenderen (d. h. standardisierten, automatisierten) Datennutzung nicht nur technischer, sondern insbesondere auch organisatorischer und prozessualer Natur: Einer Kultur verantwortungsbewusster Dateneignerschaft insbesondere der Universitätskliniken im engen Austausch von Versorgung und Forschung, auch über einzelne Fachbereiche hinweg, steht immer noch eine grundlegende Verunsicherung entgegen, die v. a. mehr Transparenz und Vertrauen im Rahmen geeigneter Strukturen und Prozesse erfordert [6].

Um in diesem Sinne neue Prozesse und Strukturen einzuführen und zu etablieren, arbeiten die DIZ an den Standorten auf strategischer Ebene eng mit Vorstand und Dekanat zusammen sowie auf operativer Ebene insbesondere mit der IT, den Informationssicherheits- und den behördlichen Datenschutzbeauftragten sowie den Ethikkommissionen.

In diesem Beitrag werden die Hauptsäulen der DIZ vorgestellt. Es wird eingegangen auf die Organisation eines DIZ, dessen organisatorische Einbettung und Interaktion mit weiteren Beteiligten und Strukturen am Standort, auf seine technische Infrastruktur und die Einbettung in der lokalen IT-Landschaft sowie auf die ethisch-rechtlichen Rahmenbedingungen. Anschließend werden die verschiedenen Services vorgestellt, die sowohl für den eigenen Standort als auch für externe Forschende angeboten werden. Abschließend wird die Rolle des DIZ als Schnittstelle nach innen und außen zur Stärkung der Kooperation und Kollaboration von unterschiedlichen (medizinischen) Fachgebieten auch über verschiedene Standorte dargelegt. Somit bilden die DIZ eine Brücke zwischen Einzelprojekten wie großen Initiativen/Konsortien, aber auch zur forschenden Gesundheitsindustrie/-wirtschaft.

## Die Datenintegrationszentren

Die Voraussetzungen an den derzeit 38 universitären sowie 3 neuen, nichtuniversitären Standorten [7] sind ganz unterschiedlich. Somit unterliegen die DIZ Standortspezifika in der Ausgestaltung auf verschiedenen Ebenen. Dazu gehören zum einen organisatorische Voraussetzungen, wie bereits existierende Infrastrukturen und IT-Systemlandschaften, Krankenhausinformationssysteme (als soziotechnisches Gesamtsystem gesehen) sowie personelle und technische Ressourcen, zum anderen die strategische Ausrichtung an den Zielen und Visionen des eigenen Standorts, auch im Sinne der Nachhaltigkeit und Verstetigung. Entscheidend für die Leistungsfähigkeit des jeweiligen DIZ ist dabei die lokale Einbindung in die Digitalisierungsstrategie des Standortes im Sinne der umfassenden Erschließung der Daten aus Versorgungs- und ggf. Forschungssystemen mit dem Ziel, zukünftige Forschungsfragen – im Sinne der „datengetriebenen Forschung und Versorgung“ – mit der entsprechenden Datenqualität und -verfügbarkeit zu adressieren.

**Abb. 1:**
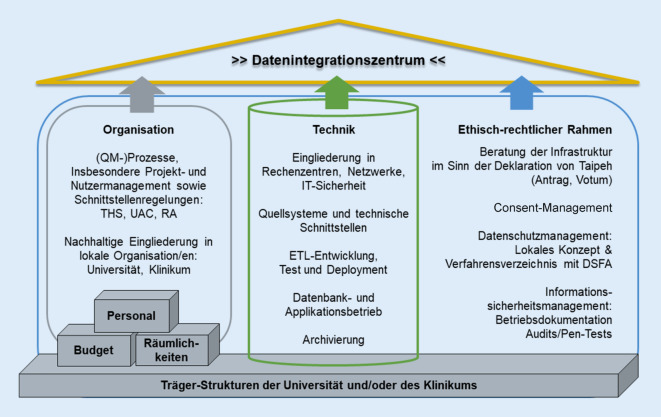
Die 3 „Säulen“ eines Datenintegrationszentrums (*DIZ*): Organisation, Technik und ethisch-rechtlicher Rahmen. (Quelle: eigene Abbildung. *DSFA* Datenschutzfolgenabschätzung, *ETL* Extract, Transform, Load (3-phasiger Prozess, bei dem Daten extrahiert, transformiert und abgelegt/gespeichert werden), *IT* Informationstechnik, *QM* Qualitätsmanagement, *RA* Rechtsabteilung, *THS* Treuhandstelle, *UAC* Use and Access Committee)

Über die vom Nationalen Steuerungsgremium (NSG) der MII etablierten Governance-Strukturen (Arbeitsgruppen, Taskforces – siehe weitere Artikel im Themenheft) werden jedoch für alle DIZ deutschlandweit die Anschlussfähigkeit sowie die Gewährleistung der Nutzung und des Austauschs von Daten und Bioproben für gemeinsame und standortübergreifende Forschungsvorhaben sichergestellt. Dies umfasst zum einen technische Aspekte, einschließlich der Gewährleistung der syntaktischen und semantischen Interoperabilität, zum anderen aber auch die (datenschutz-)rechtlichen und organisatorischen Aspekte, die es den DIZ ermöglichen, unter Verwendung von gemeinsam etablierten einheitlichen Grundprozessen die Verarbeitung, Nutzung und Auswertung von Daten und Proben(daten) aus der klinischen Routine zu unterstützen. Abb. 1 zeigt diese Aspekte als die wichtigsten Säulen eines DIZ. Dementsprechend wurden zum Abschluss der ersten Förderphase bereits im Jahr 2021 Prozesse und Ergebnisse aus diesen 3 Bereichen in einem externen Audit geprüft [8]. Darauf basierend wird aktuell eine „DIZ-Checkliste“ auch zum Onboarding neuer DIZ-Standorte entwickelt.

### Der ethisch-rechtliche Rahmen

In der MII wurden gemeinsame Regelungen und Strukturen für die Nutzung von Patientendaten und Biomaterialien sowie den Einsatz von Analysemethoden und -routinen geschaffen. Hierzu gehören ein umfassendes Vertragswerk [9] bestehend aus einem Daten- und Probennutzungsantrag, einem (Haupt‑)Vertragsdokument und allgemeinen Geschäfts- und Nutzungsbedingungen. Welche Organisationsstrukturen und Verfahrensabläufe für die einheitliche Daten- und Probennutzung notwendig sind und unter welchen Bedingungen die Daten- und Probennutzung stattfinden kann, ist in der übergreifenden [10] und ergänzend in der lokalen Nutzungsordnung verankert. Darüber hinaus wurde ein übergreifendes Datenschutzkonzept [11] erstellt, das übergreifende Anwendungsszenarien wie Machbarkeitsabfragen, verteilte Analysen sowie Daten- und Probenüberlassung behandelt und die Durchführung einer standortspezifischen Datenschutzfolgenabschätzung unterstützt.

Dabei ist zu beachten, dass ein lokales Datenschutzkonzept an einem DIZ-Standort, welches die konkreten datenschutzrelevanten Aspekte und Anforderungen berücksichtigt, unabdingbar ist. Insbesondere bei der Umsetzung eines fortlaufenden Datenschutzmanagements zwischen Datenschutzkonzept, Verzeichnis der Verarbeitungstätigkeiten und Datenschutzfolgenabschätzung sind lokale Rahmenbedingungen sowie landes- und bundesweit geltende Datenschutzbestimmungen zu berücksichtigen. Die frühzeitige und dauerhafte Einbindung lokaler, behördlicher Datenschutzbeauftragter und der Informationssicherheitsbeauftragten Personen am Standort hat sich daher als Best Practice bewährt.

Aufgrund der durchaus abweichenden, föderalen Ausgestaltung der Forschungsprivilegien im Rahmen der Datenschutz-Grundverordnung (DSGVO) in den Landesgesetzen stellt allerdings der sog. Broad Consent (allgemeine Patienteninformation und -einwilligung nach Standard der MII) eine wichtige, ergänzende, deutschlandweit einheitliche Rechtsgrundlage für die Nutzung von Daten und Bioproben für medizinische Forschungsfragestellungen dar. Dabei handelt es sich um eine national abgestimmte Patienteninformation und eine zugehörige Einwilligungserklärung, die es erlaubt, Daten und Bioproben für zukünftige medizinische Fragestellungen und Forschungsprojekte zu nutzen [12]. Auch hierbei sind spezifische lokale Anpassungen erforderlich. Die Umsetzung erfordert auch eine Lösung für das digitale Consent-Management, da die Einführung eines Broad Consent auch mit laufenden wie künftigen Studien‑/Registerprojekten in Einklang zu bringen ist.

Von den rechtlichen Rahmenbedingungen des Datenschutzes unabhängig, stellen die DIZ lokal eine „Health Database“ im Sinne der Deklaration von Taipeh [13] dar, d. h., die lokale DIZ-Organisation und Prozesse sind Gegenstand entsprechender Beratung durch die lokalen Ethikkommissionen. Dies umfasst meist sowohl die Datenintegration und -bereitstellung, die Einführung des Broad Consent als auch den Anschluss an zentrale Strukturen wie das NUM oder das Forschungsdatenportal für Gesundheit (FDPG, siehe https://forschen-fuer-gesundheit.de/), eine zentrale Anlaufstelle für Forschende, die ein Forschungsprojekt mit Routinedaten durchführen wollen.

### Die Organisation

Um einen durchgängigen Prozess vom Antrag bis zur Datennutzung (z. B. Verarbeitung und Auswertung von Daten und Proben) zu gewährleisten, sind den DIZ weitere notwendige Strukturen angegliedert. Dazu gehören die im Rahmen der übergreifenden Nutzungsordnung der MII etablierten Strukturen wie die „Use and Access Committees“ (UAC) und die Treuhandstellen (THS, Trust Center) der Standorte. Ein UAC berät eingegangene Nutzungsanträge nach organisatorischen, ggf. technischen, (datenschutz-)rechtlichen und wissenschaftlichen Gesichtspunkten und führt eine Entscheidung – gemeinsam mit weiteren Beteiligten, wie z. B. Experten aus den Fachbereichen – über die Teilnahme des Standortes an einem Nutzungsprojekt herbei. Die THS ist eine vom DIZ unabhängige Stelle, zu ihren Aufgaben können z. B. die Verwaltung von personenidentifizierenden Daten und Pseudonymen sowie von Einwilligungsinformationen bzw. Widerrufen gehören.

Bezüglich der lokalen Datenschutzkonzepte, Verfahrensverzeichnisse und Datenschutzfolgenabschätzungen sind zudem die lokalen Datenschutzbeauftragten zu involvieren. Hier können sich die Standorte aufgrund der bundeslandspezifischen Datenschutzrichtlinien und der verschiedenen Landeskrankenhausgesetze unterscheiden. Auch die Beratung durch Ethikkommissionen ist an den Standorten gemäß der MII-Nutzungsordnung vorgesehen, auch wenn die Forschungsvorhaben mit Routinedaten zumeist retrospektiv und nichtinterventionell sind.

Wichtig für den Erfolg der DIZ ist eine hohe Transparenz hinsichtlich der Ziele der MII insgesamt und der relevanten Prozesse, auch gegenüber den angegliederten und bereits existierenden Strukturen. Denn nur durch die enge Anbindung und stetige Kommunikation mit existierenden Strukturen wie den Ethikkommissionen und den Beauftragten für Datenschutz kann auch prozessseitig sichergestellt werden, dass die für die Zukunft zu erwartenden großen Mengen an Datennutzungsanfragen über das zentral etablierte FDPG bewältigt werden können. Das FDPG stellt die zentrale Kontaktstelle für alle Forschenden dar, um standortübergreifend Machbarkeitsanfragen und Forschungsanfragen, inklusive verteilter Analysen von Daten, durchzuführen. Um sicherzustellen, dass die Daten und Bioproben für genehmigte Anträge in Koordination mit dem FDPG und den teilnehmenden Standorten zusammengeführt werden, sind ferner Datenmanagementstellen (DMSt) notwendig. Der für einen Antrag zuständigen DMSt obliegt es auch, soweit notwendig, in den (Teil‑)Datensätzen enthaltene Pseudonyme mit implizitem Standortbezug durch eine einheitliche Kennzeichnung zu ersetzen sowie nach Abschluss der Datenlieferung eine Archivierung sicherzustellen. Diese Funktion der DMSt wird bis dato auch von bestimmten DIZ zusätzlich erbracht.

### Die Technik

Die an die DIZ gestellten Anforderungen führen zu einem Bedarf an spezifischer Infrastruktur, sowohl in der Infrastrukturbasis (Server, Speicher, Netzwerk) als auch in den Anwendungskomponenten. Dieser Bedarf ergibt sich u. a. daraus, dass umfangreiche medizinische Daten in einem für die DIZ definierten Format – dem MII-Kerndatensatz (KDS) bestehend aus Basis- und Erweiterungsmodulen – bereitzustellen sind (siehe Artikel von Ammon et al. in diesem Themenheft). Auch die Nutzung dieser MII-Kerndaten über die zentralen Infrastrukturen der MII benötigt eine technische Integration, die z. B. für Machbarkeitsabfragen den sicheren Zugriff über das FDPG auf die MII-Kerndaten der DIZ ermöglicht. Neben den für den KDS relevanten Daten arbeiten die DIZ teilweise auch mit weiteren Datenkategorien, wie z. B. Abrechnungs‑, Studien- und Registerdaten.

Die DIZ verfolgen einen föderierten Ansatz der lokalen Datenspeicherung, d. h., die durch die DIZ KDS-konform aufbereiteten Daten verbleiben dezentral in den DIZ. Über die technische Anbindung der DIZ an das FDPG können diese verteilt gespeicherten Daten zusammenhängend abgefragt werden. Zur Anbindung der DIZ-Infrastruktur an das FDPG gehören mehrere Komponenten, mit denen die Einhaltung von Datenschutz- und Sicherheitsvorgaben umgesetzt wird. Das sind u. a. Pseudonymisierungs‑, Verschlüsselungs- und Überwachungswerkzeuge.

**Abb. 2:**
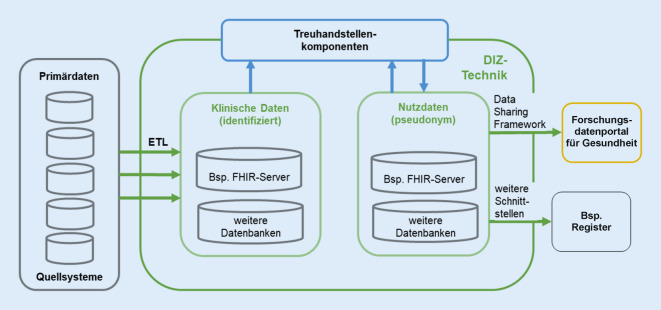
Schematische, generische Darstellung der Komponenten eines Datenintegrationszentrums (*DIZ*) für die harmonisierte Datenhaltung und -bereitstellung. (Quelle: eigene Abbildung. *ETL* Extract, Transform, Load, *FHIR* Fast Healthcare Interoperability Resources)

Abb. 2 gibt einen Überblick über die Anwendungskomponenten der DIZ, die für die harmonisierte Datenhaltung und -bereitstellung benötigt werden. Im Folgenden wird auf einige davon näher eingegangen. Komponenten, z. B. für das Management von Datennutzungsprojekten, werden nicht näher erläutert.

Ausgewählte Anwendungskomponenten der DIZ:

- Als *Primärdatenquellen* werden hier die Anwendungssysteme und Datenbanken der Patientenversorgung bezeichnet, aus denen die DIZ diejenigen Daten extrahieren, die dann integriert und in konsolidierter Form für die Datennutzungsprojekte bereitgestellt werden. Oft werden auch Data Warehouses der Kliniken als zusätzliche Quellen für die DIZ-Daten verwendet. An manchen Standorten haben die DIZ mit ihren Diensten und Schnittstellen die Verantwortung für die Data Warehouses übernommen oder tragen wesentlich zu deren Betrieb bei.
- Für die Anbindung z. B. an die MII-Strukturen wie das FDPG sind *FHIR-Server* (Fast Healthcare Interoperability Resources) die zentralen DIZ-Komponenten für die Bereitstellung von Daten. Dort werden die entsprechend der MII-KDS-Spezifikation aufbereiteten Daten aus den Primärdatenquellen gespeichert. Separate Server nehmen diejenigen Daten auf, die unter Nutzung der Trust-Center-Komponenten erzeugt werden. Für die Anbindung an weitere Forschungsverbünde sowie für bestimmte lokale Anforderungen kann das Vorhalten von Daten in *weiteren Datenbanken* mit anderen Datenformaten als FHIR notwendig sein. Manche DIZ führen aufgrund lokaler oder landesspezifischer Daten keine oder nur wenige nichtpseudonymisierte Daten.
- Die *Trust-Center-Komponenten* (Treuhandstellen-Komponenten) gewährleisten das Identitäts- und Pseudonymmanagement sowie die Verwaltung und Bereitstellung von Einwilligungsinformationen (Consent-Management). Dabei werden Daten für die Nutzung projektspezifisch gefiltert (ausgewählt) und pseudonymisiert (codiert).
- Das *Data Sharing Framework (DSF)* ist eine in der MII entstandene Middleware zur Kommunikation der DIZ untereinander und mit anderen Partnern. Ein wesentlicher zentraler Partner ist das FDPG der MII.

Beim Betrieb und der Weiterentwicklung der DIZ-Komponenten bestehen Herausforderungen u. a. durch:

- regelmäßige Aktualisierungen der MII-KDS-Spezifikationen,
- Forschungsverbünde, die andere Daten- und Integrationsstrukturen festgelegt haben,
- unterschiedliche regulatorische Rahmenbedingungen der Bundesländer, die bzgl. der Implementierung der Trust-Center-Komponenten und ggf. auch bestimmter Schnittstellen relevant sind.

### Die DIZ-Services für interne wie externe Datenproduzenten und -nutzer

Die DIZ bieten verschiedene Services sowohl für den jeweiligen lokalen Standort als auch für externe Forschende (z. B. via FDPG oder für die Region) an. Dieses Angebot kann von einem Standort zum anderen variieren. Der Kernprozess des DIZ besteht aus den Prozessschritten Datenextraktion, Datenintegration, Datenharmonisierung, Datenqualitätssicherung, Datenbereitstellung und Datenanalyse sowie der jeweils dazugehörigen Beratung.

Zunächst werden die klinischen Routinedaten aus den verschiedensten Subsystemen des jeweiligen Klinikums extrahiert und im Sinne des MII-KDS integriert. Anhand von Projekten werden weitere Datenkategorien durch lokale Datennutzung und Projekte am Standort oder auch national (z. B. radiologische Bilder wie im Projekt IMPETUS [14], kardiologische Anamnese und EKGs wie im Modul-3-Projekt ACRIBiS^1^) festgelegt und in das DIZ integriert.

Dabei findet eine Harmonisierung auf internationale Terminologien und Interoperabilitätsstandards statt und die Daten werden kompatibel zu bestehenden MII-Kerndatensatzmodulen in FHIR vorgehalten. Weitere Datenmodelle oder Interoperabilitätsstandards werden hierbei für lokale Zwecke oder zur Gewährleistung der Anschlussfähigkeit an weitere Projekte oder Initiativen unterstützt.

Die DIZ beraten die Forschenden bei der Vorbereitung von Datennutzungsprojekten, führen Machbarkeitsabfragen durch, prüfen die Antragsunterlagen und kommunizieren die Entscheidungen an die Forschenden. Zudem bilden sie die Geschäftsstellen der UACs. Bei positiver Entscheidung werden die Daten über die Transferstellen bereitgestellt, sofern zusätzlich ein entsprechendes Ethikvotum bzw. eine Feststellung vorliegt, dass keine Beratungspflicht besteht. Die Datenbereitstellung kann über mehrere Wege erfolgen. Für Datennutzungsprojekte kann es für die Analyse notwendig sein, dass die Daten direkt oder über eine Datenmanagementstelle (DMSt) an die Forschenden übertragen werden und damit die Standorte verlassen. Für andere Datennutzungsprojekte und als Alternative kann die Analyse mittels Methoden des „verteilten Rechnens“ erfolgen, d. h., die Daten bleiben an den Standorten und die Analysen finden lokal statt. Hierfür bieten die DIZ Anwendungen (z. B. DataSHIELD) und Umgebungen, die das verteilte Rechnen ermöglichen. Bei manchen Datennutzungsprojekten kann es sein, dass noch zusätzliche Daten erfasst werden müssen, über IT-Werkzeuge, die von den DIZ bereitgestellt werden (z. B. Electronic-Data-Capture-(EDC-)Systeme, Eingabemasken für Register).

Die im DIZ harmonisierten Daten können nun auch lokal für unterschiedlichste Zwecke von Krankenversorgung über Forschung und Lehre bis hin zur Qualitätssicherung bereitgestellt werden. Datennutzungsprojekte müssen nicht zwangsläufig wissenschaftlich motiviert sein, sondern können auch administrative Prozesse unterstützen. In der Infobox werden Services genannt, die von den DIZ in unterschiedlicher Zusammenstellung und Ausprägung angeboten werden.

**Abb. 3:**
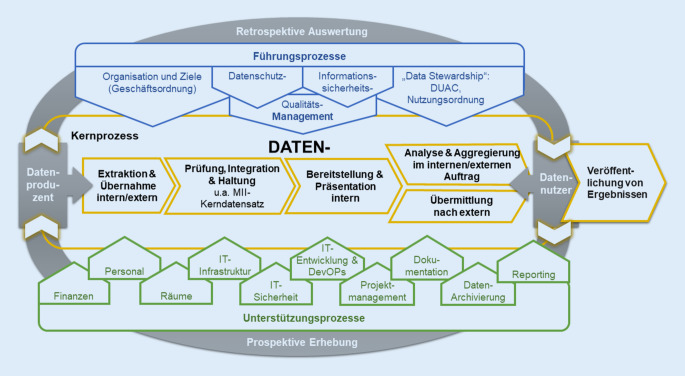
Darstellung der typischen Prozesslandschaft eines Datenintegrationszentrums (*DIZ*), mit der Datenverarbeitung als Kernprozess. (Quelle: eigene Abbildung. *DevOPs* Development/IT Operations (Zusammenarbeit von Softwareentwicklung und IT-Betrieb), *DUAC* Data Use and Access Committee, *IT* Informationstechnik, *MII* Medizininformatik-Initiative)

Abb. 3 stellt die beschriebenen Services in Form einer Prozesslandschaft eines DIZ dar, im Sinne des Qualitätsmanagements als „kundenorientierter“ Prozess zwischen Datenproduzenten und Datennutzern. Dabei werden die datenverarbeitenden Prozesse als Kernprozesse von weiteren organisatorischen und technischen Prozessen unterschieden, die sich einerseits (oben) in Management- oder Führungsprozesse und andererseits (unten) Unterstützungsprozesse aufteilen. Den Hintergrund bildet ein idealer Ablauf entlang des Datenlebenszyklus von der Datenerhebung (Datenproduzenten) zur retrospektiven Datenauswertung (Datennutzer). Um diese Kernprozesse unterstützen und die Services anbieten zu können, müssen den DIZ die entsprechenden Ressourcen bereitgestellt werden. Hierzu gehören z. B. der Zugriff auf die Quellsysteme bzw. die Bereitstellung der daraus stammenden Daten sowie die Bereitstellung von Hard- und Software zum Betrieb von klinischen Datenrepositorien, Consent-Management-Tools, aber auch Analyseplattformen. Dies kann im Sinne der dargestellten Unterstützungsprozesse ggf. dadurch gelöst werden, dass die DIZ eigene Hardware mit Virtualisierungsumgebungen betreiben bzw. Zugriff auf die entsprechende Infrastruktur des jeweiligen Universitätsklinikums und/oder der Universität erhalten.

## Fazit

Die DIZ stellen einen wichtigen Baustein in der datengetriebenen Forschung und Versorgung dar. Sie stellen ein technisches, organisatorisches und (datenschutz-)rechtliches Rahmenwerk bereit, dass es den Forschenden erlaubt, Forschungsprojekte von der Machbarkeitsabfrage bis zur Publikation in standardisierter Form, effizient, ressourcensparend und skalierbar durchzuführen. Auch für Qualitätsmanagement und Prozessoptimierung liefern die DIZ wertvolle Einblicke und Informationen.

An die Standorte der DIZ und darüber hinaus werden wachsende Anforderungen gestellt. Insbesondere muss die Digitalisierung vorangetrieben werden. Neu beschaffte IT-Systeme müssen zukunftssicher und interoperabel sein und die datengetriebene Forschung und Versorgung unterstützen. Dokumentationsprozesse müssen intuitiver werden und die Dokumentation mit intelligenten Tools optimiert werden. Den DIZ muss es ermöglicht werden, Daten in verschiedenen Formaten zu extrahieren und zu transferieren, damit sie für verschiedene Zwecke bereitgestellt werden können. Dabei dürfen Umfang und Qualität der Daten kein limitierender Faktor sein. Die Grundvoraussetzung ist, dass die Daten in digitaler Form vorliegen, was die Bedeutung des Digitalisierungsprozesses nochmals hervorhebt.

Mit den etablierten Infrastrukturen und der breiten Datenbasis, die mindestens den MII-Kerndatensatz umfasst und über spezifische Anwendungsfälle hinausgeht, sind die Grundlagen für nationale und internationale Zusammenarbeit geschaffen. Dementsprechend ist es ein Schwerpunkt der DIZ, nationale Entwicklungen, wie etwa die Einführung der elektronischen Patientenakte (ePA), aber auch internationale und europäische Initiativen wie den Europäischen Gesundheitsdatenraum (EHDS) zu berücksichtigen. Hierbei geht es um die Fortentwicklung hinsichtlich Infrastruktur und Technologie, Datenverfügbarkeit/-qualität und Interoperabilität sowie der Governance der Daten. Da die Daten in ihrer Quantität und Qualität eine entscheidende Rolle bei der Entwicklung innovativer Produkte zur Verbesserung der Patientenversorgung spielen, bieten die DIZ eine Chance für Kooperationen und Kollaborationen auch mit der Gesundheitswirtschaft [15].

### Infobox Services der Datenintegrationszentren (DIZ)

- Es besteht eine enge Kooperation mit Biobanken. DIZ betreiben Biobankinformationsmanagementsysteme und stellen diese den Biobanken zur Verfügung. Auch Biomaterialinformationen werden angeboten, sie liegen im DIZ mit den klinischen Routinedaten integriert vor.
- DIZ kooperieren mit den lokalen Studienzentren, Studienregistern und stellen Electronic-Data-Capture-(EDC-)Systeme zur standortweiten oder auch multizentrischen Nutzung bereit und integrieren diese Studiendaten ggf. später mit den klinischen Routinedaten.
- IT-Systemablösungen können vorbereitet werden, um z. B. Datenmigration in neue Krankenhausinformationssysteme und die Erhaltung historischer Patientendaten statt Datenarchivierung zu ermöglichen.
- DIZ unterstützen Controllings und Qualitätsmanagements, z. B. indem sie Hinweise im Hinblick auf Vollständigkeit, Richtigkeit und Aktualität einer Dokumentation geben. Darüber hinaus lassen sich mit den in den DIZ integrierten Daten auch weitere Services (z. B. Qualitätsberichte) anbieten.
- Es werden weitere Versorgungs- und Forschungsdienste unter Verwendung der integrierten und harmonisierten Daten angeboten, wie z. B. die Darstellungen ähnlicher Fälle zur Generierung von Erfahrungswissen im Rahmen der Krankenversorgung oder die Einbindung von fallbezogener Literatur und Leitlinien.
- Die Bereitstellung von Analyse- und Visualisierungsanwendungen oder von Plattformen für den Einsatz von Methoden der künstlichen Intelligenz gehören mittlerweile zu den angebotenen Diensten an manchen Standorten.
- Weitere Dienste, die unabhängig von der Integration von Daten angeboten werden, sind:
  - eine Kollaborationsplattform, die schon bei der Erstellung von Anträgen unterstützen kann und allen Forschenden des Standortes offensteht,
  - das Forschungsdatenmanagement für die jeweilige Universitätsmedizin oder medizinische Fakultät.
- Unterstützung von Forschenden bei Ethikanträgen. Zu beachten ist hier, dass die Erstellung von Ethikanträgen Aufgabe der antragstellenden bzw. an einem Datennutzungsprojekt beteiligten Institutionen oder Forschenden ist.

## Footnotes

1 ACRIBiS: siehe https://www.ukbnewsroom.de/acribis-personalisierte-risikobewertungen-fuer-herz-kreislauferkrankungen/. Zugegriffen: 26.02.2024.
